# USER-CENTERED DESIGN FOR SOCIALLY ASSISTIVE ROBOTIC ACTIVITIES WITH OLDER ADULTS IN LONG TERM CARE

**DOI:** 10.1093/geroni/igad104.3687

**Published:** 2023-12-21

**Authors:** Cathy Maxwell, Ritam Ghosh, Nibraas Khan, Miroslava Migovich, Judith Tate, Emily Latshaw, Lorraine Mion, Nilanjan Sarkar

**Affiliations:** Vanderbilt University, Nashville, Tennessee, United States; Vanderbilt University, Nashville, Tennessee, United States; Vanderbilt University, Nashville, Tennessee, United States; Vanderbilt University, Nashville, Tennessee, United States; The Ohio State University, Columbus, Ohio, United States; The Ohio State University, Columbus, Ohio, United States; The Ohio State University, Columbus, Ohio, United States; Vanderbilt University, Nashville, Tennessee, United States

## Abstract

Apathy is common among older adults (OAs) in long term care (LTC) and associated with adverse outcomes. Engaging residents in socially interactive multimodal activities is resource intensive. Socially assistive robots (SARs) may supplement LTC staff resources. However, few studies have involved OAs in the design of SAR activity. We conducted a multi-step user-centered design process to design and evaluate SAR activities that a) used different robots (humanoid, puppy), b) a virtual reality interface, and c) required dyadic interaction between residents. Phase 1 (system design) focused on prototype development with input from literature and an advisory panel of LTC adults and staff. Phase 2 focused on determining usability measures. Phase 3 (iterative testing) focused on field testing at two LTC sites. Multiple sessions were conducted that required looping back to earlier stages of prototype development for refinement and improvement of both the activity and the system until end users deemed them usable.

Results: 12 residents (ages 72-92, 9 women) and 5 LTC staff participated in the design of four SAR activities (music, fishing, painting, and spelling). Perceived comfort and confidence improved from baseline (d=0.708). Interaction between pairs increased from 0.16/min to 1.14/min. LTC staff were able to administer the system.

Conclusion: Older LTC adults are pivotal in informing technology development and capable of providing essential information in use and acceptance of technology. Next steps include large scale feasibility and effectiveness testing in a clinical trial among diverse older adults residing in LTC facilities.

